# Incidence, treatment variation, and survival of synchronous head and neck and esophageal carcinomas: a descriptive nationwide cohort study in the Netherlands

**DOI:** 10.1016/j.esmogo.2026.100339

**Published:** 2026-07-20

**Authors:** I.S. Moens, A.D.I. Maan, M.E. Capala, P.M. Jeene, A.D. Koch, A. Sewnaik, R.H.A. Verhoeven, S.M. Lagarde, B. Mostert, H.A. Polinder-Bos

**Affiliations:** 1 Division of Geriatric Medicine, Department of Internal Medicine, Erasmus MC, University Medical Center Rotterdam, Rotterdam, the Netherlands; 2 Department of Gastroenterology and Hepatology, Erasmus MC Cancer Institute, University Medical Center Rotterdam, Rotterdam, the Netherlands; 3 Department of Radiotherapy, Erasmus MC Cancer Institute, University Medical Center Rotterdam, Rotterdam, the Netherlands; 4 Department of Radiation Oncology, Radiotherapiegroep, Deventer, the Netherlands; 5 Department of Otorhinolaryngology – Head and Neck Surgery, Erasmus MC Cancer Institute, University Medical Center Rotterdam, Rotterdam, the Netherlands; 6 Department of Research & Development, Netherlands Comprehensive Cancer Organization (IKNL), Utrecht, the Netherlands; 7 Department of Medical Oncology, Amsterdam UMC location University of Amsterdam, Amsterdam, the Netherlands; 8 Department of Cancer Treatment and Quality of Life, Cancer Center Amsterdam, Amsterdam, the Netherlands; 9 Division of Surgical Oncology and Gastrointestinal Surgery, Department of Surgery, Erasmus MC Cancer Institute, University Medical Center Rotterdam, Rotterdam, the Netherlands; 10 Department of Medical Oncology, Erasmus MC Cancer Institute, University Medical Center Rotterdam, Rotterdam, the Netherlands

**Keywords:** esophageal neoplasms, squamous cell carcinoma of the head and neck, second primary neoplasms, incidence, therapy

## Abstract

**Background:**

Patients with head and neck squamous cell carcinoma (HNSCC) or esophageal carcinoma (EC) face elevated risk for developing second primary tumors (SPTs) in the other site. This study aimed to describe the cumulative incidence, treatment approaches and survival of these patients in the Netherlands.

**Patients and methods:**

This nationwide, retrospective cohort study used National Cancer Registry data. Patients with synchronously discovered (<6 months apart), potentially curable SPTs [M0 HNSCC and c-T1-4aN0-3M0 EC (UICC version 7 and 8)] were included (2012–2023).

**Results:**

We included 134 patients with 278 tumors (144 HNSCC, 134 EC). The cumulative incidence of SPTs was 0.46% in patients with HNSCC and 0.82% in patients with EC. Of the 103 patients (median age 66 years, 73% men) treated simultaneously (i.e., had a unified treatment plan addressing both tumors), 51.5% received treatment with curative intent, most often definitive chemoradiotherapy with carboplatin/paclitaxel (74%). Other curative treatments included radiotherapy, surgery, or radiation with cetuximab for HNSCC, and (endoscopic) resection for EC. The remaining 48.5% received palliative treatment only, mostly with radiotherapy or best supportive care. Median overall survival was 16 months (95% CI: 10-19). The 1- and 3-year survival rates were 74% and 31% for curative treatment versus 35% and 8% for palliative treatment.

**Conclusions:**

Synchronous HNSCC-EC SPTs are rare in the Netherlands, and treatment strategies vary. Despite theoretical curability, half of the patients received only palliative treatment. All patients demonstrated poor survival, regardless of the treatment approach. Multidisciplinary evaluation and individualized decision-making are essential for optimizing treatment selection in this vulnerable patient population.

## Highlights

• The incidence of potentially curable synchronously developed SPTs of the HNSCC and EC in The Netherlands is low.
• Treatment approaches vary extensively, with definitive chemoradiation for both tumors being the most common treatment with curative intent.
• Nearly half of the patients with potentially curable SPTs received exclusively palliative treatment.
• Prognosis was poor with the median survival of under 2 years being lower than in patients with one EC or HNSCC tumor.
• Pursuing curative treatment requires careful consideration of what is both feasible and desirable for each patient.

## Graphical abstract

**Figure:**
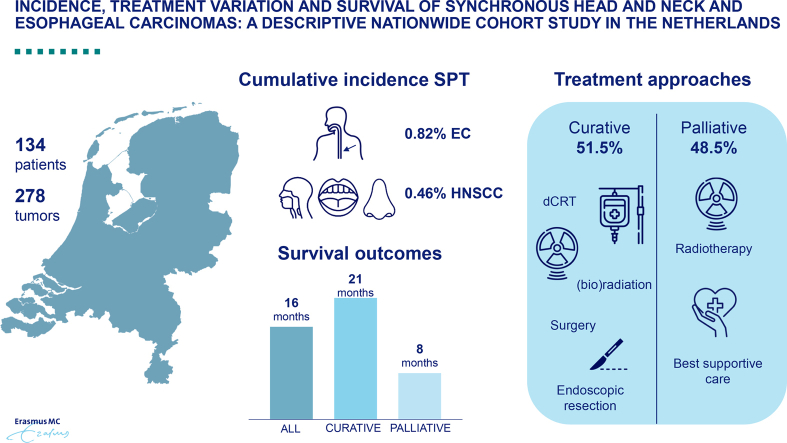

## Introduction

Head and neck squamous cell carcinoma (HNSCC) and esophageal carcinoma (EC) are malignancies with a poor prognosis.^1^^,^^2^ In 2022, HNSCC and EC were the seventh and eighth leading causes of global cancer-related mortality, respectively.^3^

Patients with HNSCC or EC share risk factors (e.g., smoking and alcohol use) and are therefore at increased risk of developing a second primary tumor (SPT) in the other anatomical site.^4^, ^5^, ^6^, ^7^, ^8^ The Warren and Gates^9^ criteria define an SPT as the presence of malignant, histologically distinct tumors, each separated by at least 2 cm. The occurrence of these SPTs in HNSCC and EC is often explained by the field cancerization theory, which suggests that prolonged exposure to carcinogens induces changes in the cells surrounding the index tumor, which may lead to the development of (pre)malignant lesions that can progress to an SPT.^10^^,^^11^

Clear treatment guidelines are available in the Netherlands for both HNSCC and EC.^12^^,^^13^ HNSCC treatment depends on the tumor stage and location. The primary treatment modalities include surgery, radiotherapy, systemic therapy, or a combination of these. Systemic therapies (e.g., chemotherapy or targeted therapy) are mainly used as radiosensitizers in HNSCC and therefore play a limited role in the curative setting.^14^ In early-stage EC, treatment typically consists of (endoscopic) resection. Treatment approaches for locally advanced esophageal squamous cell carcinoma include resection with neoadjuvant chemoradiation (nCRT) using carboplatin/paclitaxel and adjuvant nivolumab, resection with perioperative chemotherapy (cisplatin/5-fluorouracil (5-FU) or carboplatin/paclitaxel), or definitive chemoradiation (dCRT) using cisplatin/5-FU or carboplatin/paclitaxel. For locally advanced esophageal adenocarcinomas, the preferred strategy is surgical resection combined with neoadjuvant and adjuvant 5-FU, leucovorin, oxaliplatin, and docetaxel (FLOT) or resection with nCRT using carboplatin/paclitaxel and adjuvant nivolumab.^15^^,^^16^

Although clear treatment guidelines for individual tumors are present, treating patients with synchronously detected (i.e., within 6 months from each other) SPTs is more complex. Due to the limited Western data and due to the rarity of these SPTs, no established guidelines on preferred treatment approaches are currently available.

The increasing prevalence of HNSCC and EC, driven by the aging population and enhanced awareness among clinicians, underscores the need for a better understanding of current clinical care and treatment outcomes.^17^ Therefore, this study aimed to describe the nationwide cumulative incidence, the treatment approaches and survival among patients with synchronously detected, potentially curable SPTs (HNSCC and EC). With that knowledge, we aim to move closer to consensus on preferred treatment approaches and hope to improve pretreatment counseling in this often vulnerable population.^18^^,^^19^

## Materials and methods

### Patients and study design

This nationwide, retrospective, registry-based cohort study includes data from the National Cancer Registry (NCR).^20^ All patients diagnosed between January 2012 and December 2022 with synchronously detected SPTs (HNSCC and EC) were included. The 2012 start year aligns with the publication of CROSS trial, which changed EC treatment guidelines, resulting in significantly improved survival outcomes.^21^ Given the shared risk factors, squamous cell carcinomas in the head and neck and both squamous cell and adenocarcinoma in the esophagus were included.^22^ Rare tumor types with differing etiologies and treatments were excluded. Only potentially curable tumors were selected: c-T1-4aN0-3M0 esophageal tumors and M0 head and neck tumors (UICC versions 7 and 8).^23^^,^^24^ The study adhered to the Declaration of Helsinki and followed STROBE-guidelines.^25^

### Data collection

Anonymous data were collected from the NCR,^20^ including patient characteristics [sex, age, Charlson Comorbidity Index (CCI)], tobacco and alcohol use, mortality [from the Dutch Municipal Population Register (BRP) on December 31, 2024], tumor characteristics [location, histomorphology, tumor, nodes, and metastases (TNM) stage, and human papillomavirus (HPV) status for oropharyngeal tumors] and treatment type [radiotherapy, chemotherapy, targeted therapy, chemoradiation, surgery, and best supportive care (BSC)]. No data were available on details of the treatment approach such as chemotherapy regimens or radiation fields and dosages. For the TNM staging, all stages before 2017 have been re-evaluated and converted to the UICC TNM version 8. This was accomplished by individually assessing the T, N, and M stages and reclassifying them according to the version 8 criteria. For HNSCC, in case HPV status was missing, the tumor was considered HPV-negative when reclassifying the stage. Endoscopic resections included endoscopic submucosal dissection (ESD) or endoscopic mucosal resection (EMR). Esophageal resections were transthoracic esophagectomy (TTE) or transhiatal esophagectomy (THE). National CCI registration started in 2015; prior data were limited to the South of the Netherlands. Alcohol and tobacco data were collected from 2015 onward. HNSCC sites included oropharynx, hypopharynx, larynx, lips, and oral cavity.^26^ EC locations were cervical (up to 18 cm from the dental arcade), upper (18-24 cm), middle (24-32 cm), and lower (32-40 cm) esophagus. Alcohol abuse was defined as >3 units/day. All patients were treated in an expert center for HNSCC according to the Dutch guidelines.

### Study end points

The primary end points were the cumulative incidence of synchronous, potentially curable SPTs (HNSCC and EC) in all patients, and evaluation of treatment approaches and survival among simultaneously treated patients. “Simultaneously treated” refers to patients treated following SPT diagnosis, for whom a unified treatment plan was developed to address both tumors. Only these patients were analyzed, as our research focused on treatment approaches initiated in awareness of SPTs.

### Statistics

Continuous data are presented as mean ± SD or median with interquartile range (IQR), as appropriate. Categorical variables are presented as numbers and percentages. Overall survival was presented via Kaplan–Meier curves, stratified by treatment category (curative versus palliative) and tumor stage (HNSCC and EC). Survival distributions were compared for exploratory purposes using log-rank tests, and further analyzed using a Cox proportional hazards regression adjusting for age, sex, and tumor stage. Median follow-up time was estimated using the reverse Kaplan–Meier method, treating surviving patients as events. Sensitivity analyses were conducted to examine differences in characteristics between patients treated with curative intent versus those treated with palliative intent. Additionally, we assessed the impact of missing data by comparing patients with missing data for all key variables (CCI, tobacco use, and alcohol use) to those with data available for at least one of these variables. Continuous data were analyzed using independent *t*-tests and categorical data using chi-squared tests or Fisher’s exact tests in cases of low-expected cell counts. The two-sample Wilcoxon rank sum test was used when comparing medians. A *P* value < 0.05 was considered as statistically significant. Analyses were performed using Stata/SE 17.0.

## Results

### Patient characteristics

Between January 2012 and January 2023, 134 patients were diagnosed with 278 potentially curable, synchronously detected tumors (*n* = 144 HNSCC, *n* = 134 EC). Of these 134 patients, 103 received simultaneous treatment (ST). As presented in Table 1, the majority were men (72.8%), the median age was 66 years [IQR 60-71], 42.7% of them were current smokers, and more than half (53.4%) were actively using alcohol. For 30.1% of the patients, data on tobacco and alcohol consumption were missing. The median time to SPT diagnosis was 22 days [IQR 12-38], and the median time to first treatment after diagnosis of the index primary tumor was 52 days [IQR 40-67]. In the not-simultaneously treated patients (n-ST, *n* = 31), the median time to SPT diagnosis was longer [64 days (IQR 50-130)] and the median time to first treatment after diagnosis of the index tumor was shorter [23 days (IQR 20-51)] compared with patients with ST. In both patient groups, most tumors were located in the oropharynx for HNSCC (ST 47.2%, n-ST 41.7%). The majority of EC tumors were squamous cell carcinomas (ST 78.6%, n-ST 71.0%), and the most common tumor location was the middle third of the esophagus (ST 38.8%, n-ST 38.7%). In the ST group, most HNSCC were stage 4 [*n* = 54 (50%)], whereas in the n-ST group most were stage 1-2 [*n* = 19 (52.8%)]. In both groups, the majority of ECs were stage 1-2. In Supplementary Table S1 the patients with available data for all or at least one of the key variables (CCI, smoking, alcohol; *n* = 82) are compared to the patients with all key variables missing (*n* = 21).

**Table 1:** Baseline characteristics of the patients with synchronously developed HNSCC and EC with or without ST

	Patients with synchronously developed SPTs with ST (N = 103)	Patients with synchronously developed SPTs without ST (N = 31)
**Age, years (median [IQR])**	66 [60-71]	65 [58-70]
**Sex, male, *n* (%)**	75 (72.8)	26 (78.8)
**CCI, weighted (%)**		
Score 1-2 (mild)	36 (35.0)	6 (19.4)
Score 3-4 (moderate)	32 (31.1)	9 (29.0)
Score ≥5 (severe)	9 (8.7)	4 (12.9)
Missing	26 (25.2)	12 (38.7)
**Smoking status, *n* (%)**		
Current smoker	44 (42.7)	14 (45.2)
Former smoker	23 (22.3)	3 (9.7)
Never smoked	5 (4.9)	1 (3.2)
Missing	31 (30.1)	13 (41.9)
**Alcohol use, *n* (%)**		
Current or former alcohol abuse	29 (28.2)	6 (19.4)
Current alcohol use	26 (25.2)	8 (25.8)
Former alcohol use	12 (11.7)	0 (0.0)
Never used alcohol	5 (4.9)	3 (9.7)
Missing	31 (30.1)	14 (45.2)
**Median time to SPT diagnosis (days [IQR])**	22 [12-38]	64 [50-130]
**Median time to first treatment (decision) after diagnosis index tumor (days [IQR])**	52 [40-67]	23 [20-51]
**Tumor site HNSCC, *n* (%)**	∗	†
Lip	0 (0.0)	2 (5.6)
Oral cavity	9 (8.3)	14 (38.9)
Oropharynx	51 (47.2)	15 (41.7)
HPV-negative	26 (51.0)	4 (26.7)
HPV-positive	3 (5.9)	2 (13.3)
Missing	22 (43.1)	9 (60.0)
Hypopharynx	30 (27.8)	4 (11.1)
Larynx	14 (13.0)	1 (2.8)
Other^a^	4 (3.7)	0 (0.0)
**Tumor site EC, *n* (%)**		
Cervical^b^	2 (1.9)	3 (9.7)
Upper 1/3^c^	31 (30.1)	4 (12.9)
Middle 1/3	40 (38.8)	12 (38.7)
Lower 1/3	25 (24.3)	10 (32.3)
Overlapping	3 (2.9)	1 (3.2)
Not specified	2 (1.9)	1 (3.2)
**Tumor type EC, *n* (%)**		
Squamous cell carcinoma	81 (78.6)	22 (71.0)
Adenocarcinoma	13 (12.6)	8 (25.8)
Not specified	9 (8.7)	1 (3.2)
**Tumor stage TNM HNSCC, *n* (%)**	∗	†
Stage 1-2	41 (38.0)	19 (52.8)
Stage 3	13 (12.0)	6 (16.7)
Stage 4	54 (50.0)	11 (30.6)
**Tumor stage TNM EC, *n* (%)**		
Stage 1-2	55 (53.4)	27 (87.1)
Stage 3	47 (45.6)	4 (12.9)
Stage 4	1 (1.0)	0 (0.0)

CCI, Charlson Comorbidity Index; EC, esophageal carcinoma; HNSCC, head and neck squamous cell carcinoma; IQR, interquartile range; SCC, squamous cell carcinoma; SPT, second primary tumor; ST, simultaneous treatment; TNM, tumor, nodes, and metastases.

a Primary tumor unknown but SCC in neck gland.

b Tumors up to 18 cm from the dental arch.

c Tumors 18-24 cm from the dental arch.

∗ As several patients were diagnosed with multiple HNSCC, these percentages were calculated for the total number of HNSCC (n = 108) instead of the total number of patients.

† As several patients were diagnosed with multiple HNSCC, these percentages were calculated for the total number of head and neck squamous cell carcinomas (n = 36) instead of the total number of patients.

### Cumulative incidence

We calculated the cumulative incidence of the synchronously developed, potentially curable SPTs (HNSCC + EC) from 2012 to 2022 in the Netherlands. In total, 29 359 patients with potentially curable HNSCCs were diagnosed, of which 134 had a potentially curable esophageal SPT (0.46%). Furthermore, 16 376 potentially curable patients with EC were diagnosed, of which 134 had one or more potentially curable SPT(s) in the head and neck region (0.82%).

### Treatment approaches

**Figure 1:**
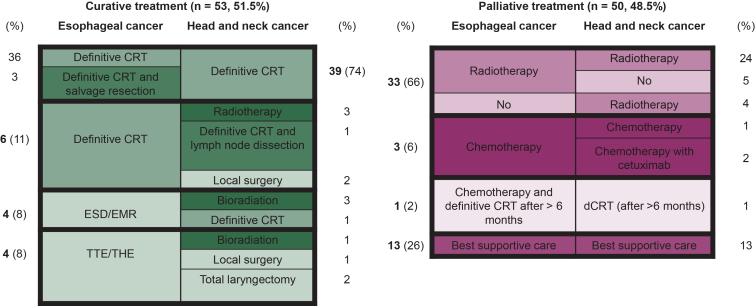
**The different treatment approaches for patients with synchronously developed HNSCC and EC. Curative approach (left) and palliative approach (right) with number of patients (%) per treatment group.** dCRT, definitive chemoradiation; EC, esophageal carcinoma; EMR, endoscopic mucosal resection; ESD, endoscopic submucosal dissection; HNSCC, head and neck squamous cell carcinoma; THE, transhiatal esophagectomy; TTE, transthoracic esophagectomy.

Figure 1 shows the treatment approaches in the 103 patients with ST. Curative treatment (*n* = 53, 51.5%) included:

• dCRT with or without salvage resection of the esophagus;
• dCRT with treatment variation for the HNSCC consisting of radiotherapy (*n* = 3), lymph node dissection combined with dCRT (*n* = 1), local resection (*n* = 1), or laser excision (*n* = 1);
• endoscopic resection (ESD or EMR) with either radiation with cetuximab (*n* = 3) or dCRT (*n* = 1) for the HNSCC;
• esophageal surgical resection (TTE or THE) with laser excision (*n* = 1), laryngectomy (*n* = 2), or radiation with cetuximab (*n* = 1) for the HNSCC. The majority of the patients in the curatively treated group received dCRT without salvage resection of the esophagus (*n* = 36, 67.9%). Chemotherapy in dCRT most often consisted of carboplatin/paclitaxel (*n* = 32), six patients received cisplatin only and one patient received carboplatin/paclitaxel combined with 5-FU.

In the palliative group (*n* = 50, 48.5%), four different treatment approaches were observed:

• radiotherapy for EC and/or HNSCC (*n* = 33, 66.0%);
• BSC (*n* = 13, 26.0%, of whom one received an esophageal stent);
• chemotherapy for EC and/or HNSCC (*n* = 3, 6.0%);
• chemotherapy with carboplatin/paclitaxel followed by chemoradiation after more than 6 months (*n* = 1, 2.0%).

Most patients received BSC because of their clinical condition and comorbidities (*n* = 9, 69.2%); in the remaining 30.8%, the decision was influenced by patient preference. Data regarding the rationale for palliative treatment (other than BSC) were unavailable. In Supplementary Table S2 (available at https://doi.org/10.1016/j.esmogo.2026.100339), patient and tumor characteristics of those treated with curative intent and those treated with palliative intent are compared. Only a statistically significant difference in EC tumor type was observed. Although the prevalence of squamous cell carcinomas (SCCs) was comparable between both groups (79% versus 78%), the curatively treated patients were diagnosed more often with adenocarcinomas (ACs) compared with the palliatively treated patients (19% versus 6%, *P* = 0.01). The palliatively treated patients more often had an unspecified EC tumor type (16%) compared with the curatively treated patients (2%).

### Survival

**Figure 2:**
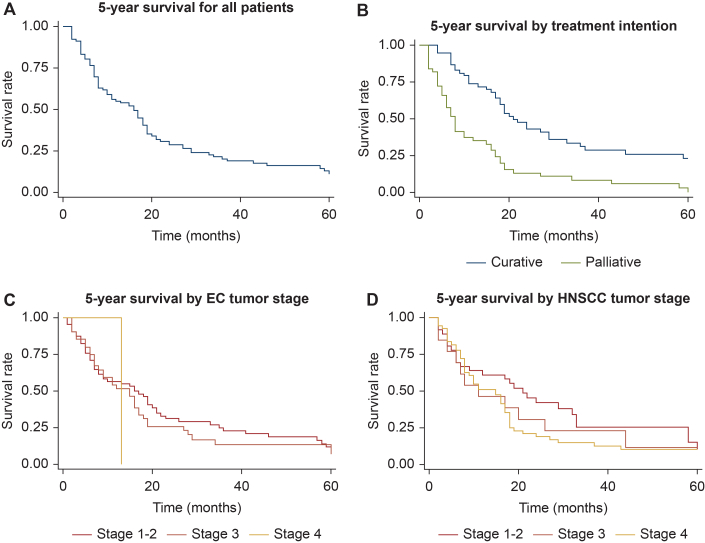
**Kaplan–Meier survival analysis of (A) all patients; (B) by treatment intention curative or palliative*P* < 0.001; (C) by EC tumor stage, *P* = 0.62; and (D) by HNSCC tumor stage, *P* = 0.23.** EC, esophageal carcinoma; HNSCC, head and neck squamous cell carcinoma.

Figure 2 shows the overall survival curves for all patients (Figure 2A), stratified by treatment intention (curative or palliative) (Figure 2B), and stratified by tumor stage for both EC (Figure 2C) and HNSCC (Figure 2D). The median overall survival for all 103 patients was 16 months (95% CI: 10-19), with a median follow-up time of 70 months (95% CI: 43-74). Patients who received treatment with curative intent had a median survival time of 21 months (95% CI: 17-29) and those who received palliative treatment had a median survival time of 8 months (95% CI: 6-12), *P* < 0.001. The 1- and 3-year survival for curative versus palliative treatment was 73.6% versus 35.0% and 31.2% versus 8.2%, respectively. Furthermore, the median survival was calculated by tumor stage. For EC, the median survival time was 18 months (95% CI: 9-21) for stage 1-2 and 12 months (95% CI: 7-17) for stage 3. No median survival was calculated for stage 4, because only one patient was included. For the HNSCC, the median survival was 21 months (95% CI: 12-29) for stage 1-2, 11 months (95% CI: 4-26) for stage 3, and 15 months (95% CI: 8-17) for stage 4 tumors. Using exploratory analysis, we compared survival distributions within EC and HNSCC tumor stages and found no significant differences between the stages (EC *P* = 0.62, HNSCC *P* = 0.23). Furthermore, an explorative Cox proportional hazards regression was performed to evaluate the association between treatment intent (curative versus palliative) and overall survival, adjusting for age, sex, and tumor stage of both EC and HNSCC. This analysis showed that patients receiving treatment with palliative intent had more than 2.5 times the risk of dying at any point in time compared with those treated with curative intent (HR 2.74, 95% CI 1.73-4.32, *P* < 0.001; Supplementary Table S3, available at https://doi.org/10.1016/j.esmogo.2026.100339). Lastly, survival curves between subgroups stratified by the combined tumor stages of EC and HNSCC were compared (Supplementary Figure S1, available at https://doi.org/10.1016/j.esmogo.2026.100339). Low stage was defined as stage 1-2 disease, and high stage as stage 3-4 disease. In total, four groups were made (both low stage versus EC high and HNSCC low versus EC low and HNSCC high versus both high stage). No statistically significant differences in survival were observed between the groups (*P* = 0.21).

## Discussion

This study describes the cumulative incidence, treatment approaches, and survival of patients with potentially curable synchronous head and neck and esophageal SPTs in the Netherlands. The cumulative incidence of SPTs in these regions was higher among patients with EC (0.82%) compared with those with HNSCC (0.46%). Furthermore, although only patients with potentially curable tumors were included, nearly half (48.5%) received palliative treatment from diagnosis. Prognosis remained poor regardless of treatment strategy, with a median survival of <2 years in both groups.

### Cumulative incidence

We found a substantially lower SPT cumulative incidence of HNSCC and EC in the Netherlands than in previous studies, which may be attributed to differences in inclusion criteria, as we included only synchronous potentially curable SPTs.^27^, ^28^, ^29^, ^30^ The recently published (2025) systematic review and meta-analysis by Maan et al.^31^ presented a pooled prevalence of 5.3% for esophageal SPTs in non-Asian populations with HNSCC. However, they included all tumor stages, as well as both synchronously and metachronously developed SPTs. These broader inclusion criteria could account for the observed higher incidence. Furthermore, Maan et al. included studies from 1988, whereas our study only included patients diagnosed from 2012 onward, which may explain differences in the reported incidence. Regarding the most recent report (2022) of the Netherlands Comprehensive Cancer Organization (IKNL) on the current and future trends of various cancers in the Netherlands, downward trends in the incidence of HNSCC and EC (the squamous cell type) have been observed for more than a decade.^17^ This decrease is most likely attributable to reduced tobacco use and alcohol consumption in the Dutch population.^7^^,^^22^

In addition, we observed a higher cumulative incidence of SPTs in patients with EC compared with patients with HNSCC. This finding may be partly explained by routine diagnostic procedures: all EC patients typically undergo gastroscopy, which could lead to incidental detection of asymptomatic, early-stage HNSCC. Given that our inclusion criteria focused on potentially curable tumors, it is plausible that early-stage HNSCCs are more likely to be identified in patients with EC through this systematic examination than vice versa. For both tumor types, the decision to routinely include positron emission tomography–computed tomography in the diagnostic workup is guided by institutional protocols rather than national guidelines.^12^^,^^13^ In line with this reasoning, various recent articles advocate for implementing endoscopic screening in patients diagnosed with HNSCC to facilitate earlier detection of esophageal SPTs.^6^^,^^7^^,^^31^^,^^32^ However, in our cohort, the majority of patients (67.9%) were first diagnosed with HNSCC, with EC being identified subsequently. This suggests that factors beyond diagnostic procedures may contribute to the observed incidence pattern, which warrants further investigation.

### Treatment approaches

Literature on the treatment of patients with synchronously developed HNSCC and EC is limited and all available studies originate from Asian countries. A recently published Japanese study by Ueda et al.^33^ described treatment approaches in patients with synchronously developed HNSCC and EC SPTs and found that only 9.4% received ST, which is substantially lower than the 76.9% who underwent ST in our cohort. CRT for HNSCC combined with endoscopic resection for EC was described as the most frequent non-ST approach. Another Japanese study, by Morita et al.,^34^ explains that simultaneous dCRT for SPTs is controversial in Japan due to the larger radiation field and associated toxicity.^35^ Simultaneous surgery is the “gold standard” in Japan; however, it is often avoided due to its complexity and invasiveness. Both the Japanese study by Morita et al.^34^ and the Korean study by Park and Lee^36^ reported, in line with our findings, that dCRT was the most common ST approach for synchronously discovered SPTs. The dCRT comprised mostly of cisplatin/5-FU in contrary to our study where patients received mostly carboplatin/paclitaxel. Although the carboplatin/paclitaxel schedule deviates from the ESMO guidelines, it aligns with the Dutch national guideline, which endorses this combination based on the CROSS trial results (demonstrating comparable survival outcomes with improved tolerability) and extensive national clinical experience.^12^^,^^15^^,^^21^ Nevertheless, Morita et al. show that simultaneous therapy—both dCRT and surgery—is feasible and effective. Notably, <5% of the patients in their National Cancer Center received BSC, a proportion that is significantly lower than in our population. Unfortunately, their study provides no information on the rationale behind choosing BSC. As previously mentioned, our study lacks data on the decision-making incentives as well, which limits our ability to determine why nearly half of the patients with potentially curable tumors received palliative treatment. Patient and tumor characteristics were compared between the patients treated with curative intent and patients treated with palliative intent (Supplementary Table S2, available at https://doi.org/10.1016/j.esmogo.2026.100339). Although a statistically significant difference in EC tumor type was observed, this finding does not account for why nearly half of the patients were treated with palliative intent, given that the prevalence of squamous cell carcinoma is comparable between groups. We did observe a difference in CCI score, where patients receiving curative treatment have a lower CCI of 1-2 (42% versus 28% in the palliative group, *P* = 0.42). Although this difference holds no statistical significance, the palliatively treated patients could have already been more frail before treatment. Furthermore, the data we do have on treatment decision-making showed that one-third of the decisions for BSC could be explained by patient preference. Additionally, no data were available on considerations regarding the technical feasibility of the treatments. We hypothesize that technical feasibility may have influenced the decision-making process, as the combination of tumors likely necessitates treatment of an expanded anatomical field.

### Survival

Our study demonstrated a poor median survival of 16 months among patients with these SPTs. This is shorter than the median survival of 20 months in patients with nonmetastatic EC in the Netherlands.^37^ In 2017, Chen et al.^38^ used propensity score matching to compare outcomes between patients with synchronous HNSCC and esophageal squamous cell carcinoma (ESCC), and those with isolated ESCC. The SPT group had a significantly worse prognosis, with a median survival of 13.5 months compared with 17.2 months for the isolated ESCC group. Although specific NCR survival data for the subgroup of potentially curable HNSCC are not available, survival data for the overall HNSCC population are. While survival is typically reported by anatomical subsite, we used aggregated data for comparison, consistent with our pooled analysis. The NCR data across all tumor stages show substantially better survival than observed in our cohort, with a median survival ranging between 5 and 10 years depending on the anatomical site (excluding HNSCC sites not represented in our data). These findings highlight the worse prognosis associated with SPTs compared with patients diagnosed with solely one tumor. Previous studies have demonstrated that HPV-related oropharyngeal carcinomas are associated with improved survival compared with other head and neck cancers.^39^^,^^40^ However, evidence from studies specifically focusing on SPTs suggests that patients with HPV-unrelated HNSCC have a higher risk of developing SPTs and experience worse survival outcomes.^41^ This may partly explain the higher proportion of HPV-negative tumors observed in our cohort of patients with SPTs. Nevertheless, this finding should be interpreted with caution, as HPV status was missing for a substantial proportion of patients in our data set, limiting the ability to draw firm conclusions. In line with our expectations our explorative adjusted Cox proportional hazards regression showed a 2.5 times higher risk of dying at any point in time for patients treated with palliative versus curative intent. However, the potential for selection bias is considerable, given that treatment intent was not randomly assigned and that patients receiving palliative treatment were likely more frail at diagnosis. Overall, the presence of a SPT appears to be an independent negative prognostic factor for patients with HNSCC and patients with EC, which should be borne in mind during the decision-making process (both in terms of providing information to patients and deciding on treatment approach).^42^

The reduced median survival observed in patients with SPTs may be partly explained by the frequent selection of palliative over curative treatment strategies, as demonstrated in our study. As mentioned in the previous paragraph, the treatment of two primary tumors is highly intensive for patients and sometimes poses significant anatomical and technical challenges, often limiting the feasibility of curative interventions. Moreover, patients with SPTs may have a higher overall disease burden and a higher prevalence of comorbidities, potentially influenced by lifestyle-related risk factors such as tobacco and alcohol use.^43^ In our cohort, data on smoking and alcohol consumption were missing for nearly one-third of patients. Among those with available data, almost half of the patients were current smokers, and the majority reported regular alcohol consumption. Development of multiple primary tumors can reflect cumulative exposure to carcinogenic substances. Literature on tobacco and alcohol use in patients with EC and HNSCC in the Netherlands is limited. A previously conducted Dutch study (2014) on patients with HNSCC reported a higher prevalence of smoking but lower rates of alcohol consumption compared to our cohort.^44^ Furthermore, the absence of robust research and standardized treatment guidelines for SPTs likely contributes to variability in clinical decision-making and, as a consequence, to patient outcomes. Lastly, determining which tumor drives prognosis is a recurrent topic in multidisciplinary team discussions for these patients. In our analysis, survival did not differ significantly across groups defined by tumor stage combinations (low/low, high/low, low/high and high/high; Supplementary Figure S1, available at https://doi.org/10.1016/j.esmogo.2026.100339). This finding underscores the complexity of prognostic assessment, emphasizing the importance of integrated patient assessment.

### Strengths and limitations

Our study is the first to describe treatment approaches for potentially curable SPTs (HNSCC and EC) in the Netherlands, using data from the largest nationwide registry database. Our findings add important information to existing literature, which is primarily focused on Asian populations and consists of smaller cohorts compared with our study. However, several limitations need to be addressed. As this is a retrospective cohort study, we were limited to using previously collected data, which lacked information on tobacco and alcohol use, HPV status, detailed information on the treatment approaches (dose, schemes, radiation fields), the rationale behind choosing palliative or curative treatment (e.g., medical decision versus patient preference), and cause of death. In the sensitivity analysis to assess the impact of missing data (CCI, tobacco, and alcohol use), we found no relevant differences in characteristics and outcomes between both groups (Supplementary Table S1, available at https://doi.org/10.1016/j.esmogo.2026.100339). Nonetheless, given the limited available literature, our descriptive study provides valuable insights into the cumulative incidence, treatment approaches, and mortality of these rare SPTs. Lastly, to interpret the study findings, it is important to consider that we present a selection of all patients with HNSCC or EC SPT in the Netherlands, namely, those with (i) synchronously developed, (ii) potentially curable disease, and (iii) received ST. This specific selection was made, as treatment decisions are most challenging in this population.

### Clinical implications and future research

As mentioned in the introduction of this manuscript, there is a need for better understanding of current clinical care and treatment outcomes in patients with potentially curable synchronously developed SPTs of the HNSCC and EC. Our study provides an overview of treatment approaches and associated mortality, constituting an important first step toward achieving consensus on preferred treatment strategies. A European Delphi-based meeting should be conducted to establish consensus on the treatment approach for these vulnerable patients. Furthermore, treatment outcomes should be evaluated in a large prospective European cohort. These outcomes will support the development of guidelines and prognostic models to guide treatment selection. Our findings highlight the importance of integrating patient and tumor characteristics when determining treatment intent. Qualitative studies should be conducted to further investigate factors influencing the choice of palliative care (including whether it is due to the technical or anatomical infeasibility of curative treatment, patient characteristics, or the patient's preference), to determine when curative treatment should be considered in this group with exclusively potentially curable tumors. Meanwhile, the decision to pursue curative treatment requires careful consideration of what is both feasible and desirable for each patient, incorporating biological age, overall condition, and patient preferences into a tailored treatment plan. To optimize this decision-making process, we recommend discussing with patients with SPTs in a multidisciplinary setting and performing a comprehensive geriatric assessment when indicated.^45^^,^^46^

### Conclusions

The cumulative incidence of potentially curable synchronously developed SPTs of the HNSCC and EC in the Netherlands is lower than described in previous literature, and treatment approaches vary extensively. Although our cohort consisted of only patients with theoretically curable SPTs, almost half of them received palliative treatment, which highlights the gap between theoretical curability and actual treatment approach. Notably, the patients treated with curative intent, who were expected to be relatively fit, still demonstrated substantially worse survival than those with a single tumor. Our findings provide valuable insight into current clinical practice and underscore the need to improve pretreatment counseling in this often vulnerable population.
