# Supplementary material for: Incidence, treatment variation, and survival of synchronous head and neck and esophageal carcinomas: a descriptive nationwide cohort study in the Netherlands
Source: ESMO Gastrointest Oncol. 2026 Jul 20;13(Pt B):100339. doi: 10.1016/j.esmogo.2026.100339 (PMC13459566; doi:10.1016/j.esmogo.2026.100339)
Supplement: Supplementary file 1 [file mmc2.pdf]

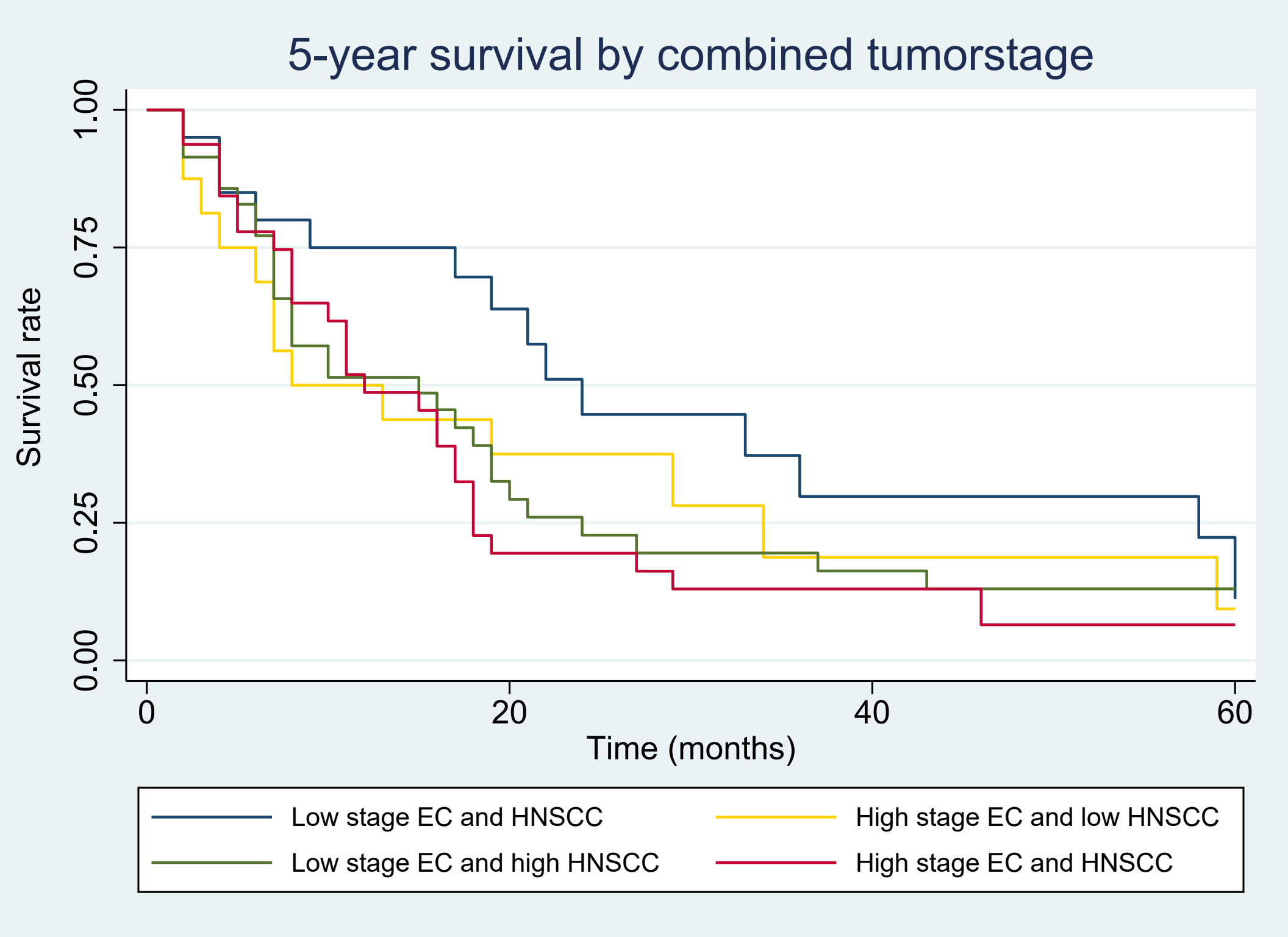

5-year survival by combined tumorstage
Survival rate
1.00
0.75
0.50
0.25
0.00
0
20
40
60
Time (months)
Low stage EC and HNSCC
High stage EC and low HNSCC
Low stage EC and high HNSCC
High stage EC and HNSCC
