# Supplementary material for: Incidence, treatment variation, and survival of synchronous head and neck and esophageal carcinomas: a descriptive nationwide cohort study in the Netherlands
Source: ESMO Gastrointest Oncol. 2026 Jul 20;13(Pt B):100339. doi: 10.1016/j.esmogo.2026.100339 (PMC13459566; doi:10.1016/j.esmogo.2026.100339)
Supplement: Supplementary file 2 [file mmc1.docx]

**Supplementary Table 1**. Characteristics of patients with synchronously developed head and neck squamous cell carcinoma (HNSCC) and esophageal carcinoma (EC) with the CCI, smoking and alcohol key variables (≥1) available compared to the patients with all key variables missing.

|  | | | Key variables (≥1) available (n=82) | All key variables missing (n=21) | P-value |
| --- | --- | --- | --- | --- | --- |
| Age, years (mean, SD) | | | 66.4 (8.6) | 64.2 (7.1) | 0.28 |
| Sex, male (n, %) | | | 61 (74.4) | 14 (66.7) | 0.48 |
| Median time to SPT-diagnosis  (days, [IQR]) | | | 22 [11-38] | 25 [13-40] | 0.42 |
| Median time to first treatment (decision) after diagnosis index tumor (days, [IQR]) | | | 50 [40-65] | 55 [41-91] | 0.23 |
| Tumor site HNSCC | | | * | ** | 0.57 |
|  | Lip | | 0 (0.0) | 0 (0.0) |  |
|  | Oral cavity | | 7 (8.2) | 2 (8.7) |  |
|  | Oropharynx | | 41 (48.2) | 10 (43.5) | 0.19 |
|  |  | HPV Positive | 3 (7.3) | 0 (0.0) |  |
|  |  | HPV negative | 26 (63.4) | 0 (0.0) |  |
|  |  | Missing | 12 (29.3) | 10 (100) |  |
|  | Hypopharynx | | 21 (24.7) | 9 (39.1) |  |
|  | Larynx | | 12 (14.1) | 2 (8.7) |  |
|  | Other ^1^ | | 4 (4.7) | 0 (0.0) |  |
| Tumor site EC | | |  |  | 0.02 |
|  | Cervical^2^ | | 1 (1.2) | 1 (4.8) |  |
|  | Upper 1/3^3^ | | 25 (30.5) | 6 (28.6) |  |
|  | Middle 1/3 | | 33 (40.2) | 7 (33.3) |  |
|  | Lower 1/3 | | 21 (24.7) | 4 (19.0) |  |
|  | Overlapping | | 12 (14.1) | 3 (14.3) |  |
|  | Not specified | | 4 (4.7) | 0 (0.0) |  |
| Tumor type EC | | |  |  | 0.09 |
|  | Squamous cell carcinoma | | 65 (79.3) | 16 (76.2) |  |
|  | Adenocarcinoma | | 8 (9.8) | 5 (23.8) |  |
|  | Not specified | | 9 (11.0) | 0 (0.0) |  |
| Tumor stage, TNM HNSCC | | | * | ** | 0.18 |
|  | Stage 1-2 | | 35 (41.2) | 6 (26.1) |  |
|  | Stage 3 | | 8 (9.4) | 5 (21.7) |  |
|  | Stage 4 | | 42 (49.4) | 12 (52.2) |  |
| Tumor stage TNM EC | | |  |  | 0.87 |
|  | Stage 1-2 | | 44 (53.7) | 11 (52.4) |  |
|  | Stage 3 | | 37 (45.1) | 10 (47.6) |  |
|  | Stage 4 | | 1 (1.2) | 0 (0.0) |  |
| Treatment choice, curative (n, %) | | | 43 (52.4) | 10 (47.6) | 0.69 |
| Survival, months (median (95% CI)) | | | 16 (11-19) | 10 (4-19) | 0.14 |

^1^ = primary tumor unknown, but SCC in neck gland | ^2^ = Tumors up to 18 cm from the dental arch | ^3^ = Tumors 18-24cm from the dental arch
*= as several patients were diagnosed with multiple HNSCC, these percentages were calculated for the total number of HNSCC for patients with data for at least one key variable (n=85) instead of the total number of patients.
**= as several patients were diagnosed with multiple HNSCC, these percentages were calculated for the total number of HNSCC for patients with data missing for all key variable (n=23) instead of the total number of patients.

**Supplementary Table 2.** Characteristics of patients with synchronously developed head and neck squamous cell carcinoma (HNSCC) and esophageal carcinoma (EC) treated with curative intent compared to the patients treated with palliative intent.

|  | | | Patients treated with curative intent (n=53) | Patients treated with palliative intent (n=50) | P-value |
| --- | --- | --- | --- | --- | --- |
| Age, years (mean, SD) | | | 64.1 (7.6) | 67.9 (8.6) | 0.38 |
| Sex, male (n, %) | | | 40 (75.5) | 35 (70.0) | 0.53 |
| CCI^1^, weighted (%) | | |  |  | 0.42 |
|  | Score 1-2 (mild) | | 22 (41.5) | 14 (28.0) |  |
|  | Score 3-4 (moderate) | | 16 (30.2) | 16 (32.0) |  |
|  | Score ≥5 (severe) | | 3 (5.7) | 6 (12.0) |  |
|  | Missing | | 12 (22.6) | 14 (28.0) |  |
| Smoking status | | |  |  | 0.28 |
|  | Current smoker | | 27 (50.9) | 17 (34.0) |  |
|  | Former smoker | | 10 (18.9) | 13 (26.0) |  |
|  | Never smoked | | 4 (7.5) | 1 (2.0) |  |
|  | Missing | | 12 (22.6) | 19 (38.0) |  |
| Alcohol use | | |  |  | 0.63 |
|  | Current or former alcohol abuse | | 17 (32.1) | 12 (24.0) |  |
|  | Current alcohol use | | 14 (26.4) | 12 (24.0) |  |
|  | Former alcohol use | | 7 (13.2) | 5 (10.0) |  |
|  | Never used alcohol | | 3 (5.7) | 2 (4.0) |  |
|  | Missing | | 12 (22.6) | 19 (38.0) |  |
| Median time to SPT-diagnosis  (days, [IQR]) | | | 23 [16-29] | 16 [8-35] | 0.13 |
| Median time to first treatment (decision) after diagnosis index tumor (days, [IQR]) | | | 41 [25-61] | 42 [26-60] | 0.76 |
| Tumor site HNSCC | | | * | ** | 0.65 |
|  | Lip | | 0 (0.0) | 0 (0.0) |  |
|  | Oral cavity | | 3 (5.3) | 6 (11.8) |  |
|  | Oropharynx | | 29 (46.2) | 22 (43.1) | 0.28 |
|  |  | HPV positive | 3 (10.3) | 0 (0.0) |  |
|  |  | HPV negative | 14 (48.3) | 12 (54.5) |  |
|  |  | Missing | 12 (41.4) | 10 (45.5) |  |
|  | Hypopharynx | | 17 (29.8) | 13 (25.5) |  |
|  | Larynx | | 6 (10.5) | 8 (15.7) |  |
|  | Other ^2^ | | 2 (3.5) | 2 (3.9) |  |
| Tumor site EC | | |  |  | 0.09 |
|  | Cervical ^3^ | | 2 (3.8) | 0 (0.0) |  |
|  | Upper 1/3^4^ | | 13 (24.5) | 18 (36.0) |  |
|  | Middle 1/3 | | 24 (45.3) | 16 (32.0) |  |
|  | Lower 1/3 | | 14 (26.4) | 11 (22.0) |  |
|  | Overlapping | | 0 (0.0) | 3 (6.0) |  |
|  | Not specified | | 0 (0.0) | 2 (4.0) |  |
| Tumor type EC | | |  |  | 0.01 |
|  | Squamous cell carcinoma | | 42 (79.2) | 39 (78.0) |  |
|  | Adenocarcinoma | | 10 (18.9) | 3 (6.0) |  |
|  | Not specified | | 1 (1.9) | 8 (16.0) |  |
| Tumor stage, TNM HNSCC | | | * | ** |  |
|  | Stage 1-2 | | 25 (43.9) | 16 (31.4) |  |
|  | Stage 3 | | 7 (12.3) | 6 (11.8) |  |
|  | Stage 4 | | 25 (43.9) | 29 (56.9) |  |
| Tumor stage TNM EC | | |  |  | 0.37 |
|  | Stage 1-2 | | 31 (58.5) | 24 (48.0) |  |
|  | Stage 3 | | 22 (41.5) | 25 (50.0) |  |
|  | Stage 4 | | 0 (0.0) | 1 (2.0) |  |

^1^ Charlson Comorbidity Index |^2^ Primary tumor unknown but SCC in neck gland |^3^ Tumors up to 18 cm from the dental arch |^4^ Tumors 18-24cm from the dental arch
* = as several patients were diagnosed with multiple HNSCC, these percentages were calculated for the total number of head and neck squamous cell carcinomas (n=57) instead of the total number of patients.
** = as several patients were diagnosed with multiple HNSCC, these percentages were calculated for the total number of head and neck squamous cell carcinomas (n=51) instead of the total number of patients.

**Supplementary Table 3**. Cox regression between treatment intent and time to death in patients with synchronously developed head and neck squamous cell carcinoma (HNSCC) and esophageal carcinoma (EC)

| Model* | Adjustment | Treatment HR (95% CI) | p-value |
| --- | --- | --- | --- |
| Model 1 | Crude | 2.82 (1.81-4.39) | <0.001 |
| Model 2 | Age, sex | 2.84 (1.80-4.47) | <0.001 |
| Model 3 | Age, sex, tumor stage EC, tumor stage HNSCC | 2.74 (1.73-4.32) | <0.001 |

*Abbreviations: HR = Hazard ration; CI = confidence interval; EC = esophageal carcinoma; HNSCC = head and neck squamous cell carcinoma*
** Reference category of treatment intent is curative*

**Supplementary Figure 1**. Kaplan-Meier survival curves for subgroups of patients with synchronously developed head and neck squamous cell carcinoma (HNSCC) and esophageal carcinoma (EC) stratified by combined tumor stage of EC and HNSCC (low stage I-II; high stage: III-IV), comprising low/low, low/high, high/low, and high/high combinations (p=0.21).

*Note: This figure is intended for color reproduction (print/online).*
